# The novel ZIP4 regulation and its role in ovarian cancer

**DOI:** 10.18632/oncotarget.21435

**Published:** 2017-09-30

**Authors:** Qipeng Fan, Qingchun Cai, Pengfei Li, Wenyan Wang, Jing Wang, Emily Gerry, Tian-Li Wang, Ie-Ming Shih, Kenneth P. Nephew, Yan Xu

**Affiliations:** ^1^ Department of Obstetrics and Gynecology, Indiana University School of Medicine, Indianapolis, IN 46202, USA; ^2^ Pharmaceutical Research Center, Beijing Chao-Yang Hospital, Capital Medical University, Beijing, 100020, P.R. China; ^3^ Department of Obstetrics and Gynecology, The Second Hospital of Anhui Medical University, Hefei City, 230601, P.R. China; ^4^ MASDINO (Beijing) Medical Research Co. Ltd., Beijing, 100123, P.R. China; ^5^ Department of Pathology, Johns Hopkins School of Medicine, Baltimore, MD 21231, USA; ^6^ Medical Sciences, Indiana University School of Medicine, Jordan Hall 302, Bloomington, IN 47405, USA

**Keywords:** ZIP4, LPA, ovarian cancer, cancer stem cells (CSC)

## Abstract

Our RNAseq analyses revealed that ZIP4 is a top gene up-regulated in more aggressive ovarian cancer cells. ZIP4's role in cancer stem cells has not been reported in any type of cancer. In addition, the role and regulation of ZIP4, a zinc transporter, have been studied in the context of extracellular zinc transporting. Factors other than zinc with ZIP4 regulatory effects are essentially unknown. ZIP4 expression and its regulation in epithelial ovarian cancer cells was assessed by immunoblotting, quantitative PCR, or immunohistochemistry staining in human ovarian tissues. Cancer stem cell-related activities were examined to evaluate the role of ZIP4 in human high-grade serous ovarian cancer cells *in vitro* and *in vivo*. RNAi and CRISPR techniques were used to knockdown or knockout ZIP4 and related genes. Ovarian cancer tissues overexpressed ZIP4 when compared with normal and benign tissues. ZIP4 knockout significantly reduced several cancer stem cell-related activities in EOC cells, including proliferation, anoikis-resistance, colony-formation, spheroid-formation, drug-resistance, and side-population *in vitro*. ZIP4-expressing side-population highly expressed known CSC markers ALDH1 and OCT4. ZIP4 knockout dramatically reduced tumorigenesis and ZIP4 overexpression increased tumorigenesis *in vivo*. In addition, the ZIP4-expressing side-population had the tumor initiating activity. Moreover, the oncolipid lysophosphatic acid effectively up-regulated ZIP4 expression via the nuclear receptor peroxisome proliferator-activated receptor gamma and lysophosphatic acid 's promoting effects in cancer stem cell-related activities in HGSOC cells was at least partially mediated by ZIP4 in an extracellular zinc-independent manner. Our critical data imply that ZIP4 is a new and important cancer stem cell regulator in ovarian cancer. Our data also provide an innovative interpretation for the apparent disconnection between low levels of zinc and up-regulation of ZIP4 in ovarian cancer tissues.

## INTRODUCTION

Epithelial ovarian cancer (EOC) is the deadliest gynecologic cancer. The Cancer Genome Atlas(TCGA) data have set the genetic landscape of EOC [1]. We have developed a highly aggressive EOC cell line (ID8-P1) through *in vivo* passage of ID8-P0 cells in C57BL6 mice [2]. The tumor/ascites formation time is reduced from ~90 days for ID8-P0 cells to ~30 days in different ID8-P1 cell lines isolated from tumors in different organs or from ascites [2]. RNAseq data in two independent pairs of ID8-P0 and ID8-P1 cells were obtained. *Zip4* is among the genes highly up-regulated in ID8-P1 vs. ID8-P0 cells.

Intracellular zinc (Zn) homeostasis is tightly regulated under physiological conditions [3]. ZIP4 is one of the Zn transporters [4]. The regulation and activities of ZIP4 have been almost exclusively studied in the context of Zn [5–7]. ZIP4 plays tumor promoting roles in many cancer types, including pancreatic cancer, hepatocellular carcinomas, breast cancer, and glioma [8–10]. In contrast, Zn levels are significantly lower in prostate and ovarian cancer tissues, when compared to normal tissues [11] and Zn induces apoptosis in prostate and ovarian cancer cells [12, 13]. However, while ZIP4 expression is down-regulated in prostate carcinoma and it has an inhibitory effect on prostate carcinoma cell proliferation and invasion, in an Zn-dependent manner,[8] ZIP4 is over-expressed in EOC tissues,[14] and the role of ZIP4 in EOC has not been reported.

ZIP4 presents in the stem cell niche and intestine integrity [15], but has not been shown as a cancer stem cell (CSC) marker/gene in any cancer type. Our group was one of the earliest to identify EOC CSC [16–19]. Various CSC markers have been identified by different research groups, including CD44, CD117 (Kit), CD133, aldehyde dehydrogenase 1 (ALDH1), Oct4, EpCAM, Nanog, Nestin, and ABCG2 [16, 19–22]. Among the most consistent markers for EOC CSC are spheroid-formation and the side-population (SP) cells (capable of excluding Hoechst 33342 from cells), [23, 24] which have been shown to be an enriched source of CSC.

We were the first to show that the bioactive lipid molecule lysophosphatic acid (LPA) is a growth factor for EOC [25–28]. Responses to LPA are mediated primarily by their plasma membrane bound G-protein coupled receptors (LPAR_1-6_) [29, 30]. In addition, LPA has been identified as a ligand for the nuclear receptor peroxisome proliferator-activated receptor gamma (PPAR*γ*) [31, 32]. The LPA-PPAR*γ* studies are mainly limited to the vascular and metabolic processes [32]. During the course of this study, Seo *et al.* have shown that autotaxin (ATX) stimulates the maintenance of EOC stem cells through LPA-mediated autocrine mechanism [33]. LPAR_1_ and AKT1 are identified as the important down-stream signaling molecules mediating these effects in Seo's work [33].

While our results are highly consistent to Seo's work in supporting LPA's CSC activity in EOC, a novel LPA-PPAR*γ*-ZIP4 and extracellular Zn-independent signaling pathway and its involvement in CSC has been revealed. Genetic, cell biological, and biochemical analyses were conducted *in vitro* and *in vivo*. CSC-related activities, including anoikis-resistance, drug-resistance, colony-formation, spheroid-formation, side-population, and tumorigenesis were the central focuses of the work.

## RESULTS

### ZIP4 and other CSC markers in EOC were over-expressed in human EOC tissues and in ID8-P1 vs. ID8-P0 cells

The TCGA and Oncomine data suggest that the *ZIP4* gene is over-expressed in EOC [14]. We confirmed the over-expression of ZIP4 in EOC using a subset of tissues obtained from CHTN, which we have used in our previous studies [34]. ZIP4 protein was over-expressed in EOC vs. benign and normal ovarian tissues (Supplementary Figure 1; representative data). We also used an ovarian cancer TMA to evaluate ZIP4 expression. The results are summarized in Supplementary Table 1. Twelve (12) of 16 (75%) of HGSOC samples expressed high levels of ZIP4. The remaining (4 of 16) HGSOC tissues also expressed ZIP4, albeit with lower levels. Only 1 of 4 (25%) low grade serous ovarian cancer tissue samples expressed a high level of ZIP4 and none of other groups of tissues (ovarian endometrioid carcinoma, serous borderline ovarian cancer, and control tissues) expressed high levels of ZIP4. Representative results are shown in Supplementary Figure 2.

RNAseq analysis [35] of two independent pairs of ID8-P0 and ID8-P1 cells revealed more than 1,000 genes up-regulated in ID8-P1 vs. ID8-P0 cells, among which, up-regulation of more than 15 genes was confirmed by Western blot analysis, ELISA, and/or RT-qPCR in at least two human HGSOC cell lines, PE04 and OVCAR3, at the mRNA and/or protein levels (Table 1 and data to be published elsewhere). Interestingly, several previously identified EOC cancer stem cell (CSC) markers, including CD44, CD24, CD117 (Kit), and EpCAM, [16] were up-regulated in ID8-P1 vs. ID8-P0 cells (Table 1). Several key signaling molecules involved in ID8 cells are also involved in the aggressiveness in human EOC cells as we showed previously [2]. ID8 cells may not fully recapitulate HGSOC characteristics, but the RNAseq data provided a guideline for potential functionally important genes. The majority of the work in this manuscript was conducted using human HGSOC cells.

**Table 1:** Genes with altered expression in the more aggressive ID8-P1 vs. less aggressive ID8-P0 cells detected by RNAseq

Gene	Fold (ID8-P1 vs. ID8-P0)	Read in ID8-P1	*P*
***Zip4***	183	1174	3.20E-217
***Piwil2***	157	569	1.55E-117
***Ncam1***	6.2	2841	2.76E-73
***Vegfa***	7.5	11016	1.38E-68
***Cyp27a1***	9.9	545	1.92E-68
***Kit (Cd117)***	43	278	3.40E-53
***Akt3***	6.7	518	3.61E-47
***Sox9***	2.6	718	1.04E-26
***Cd24a***	7.7	130	2.10E-19
***Ctnnb1 (β-catenin)***	1.6	11249	5.02E-14
***Epcam***	4.9	91	1.13E-08
***Abcc1***	1.5	2762	2.91E-08
***CD44***	1.4	1426	8.31E-06

Reads in ID8-P1 cells reflect the relative RNA expression levels in EOC cells. Fold and Read values are averages of the two pairs of the samples. *P* values for the listed genes are all <10^-5^. The order of the genes listed is based on *P* values, with the smallest on the top.

### LPA-dose and -time dependently up-regulated ZIP4 via PPARγ in EOC cells

**Figure 1:**
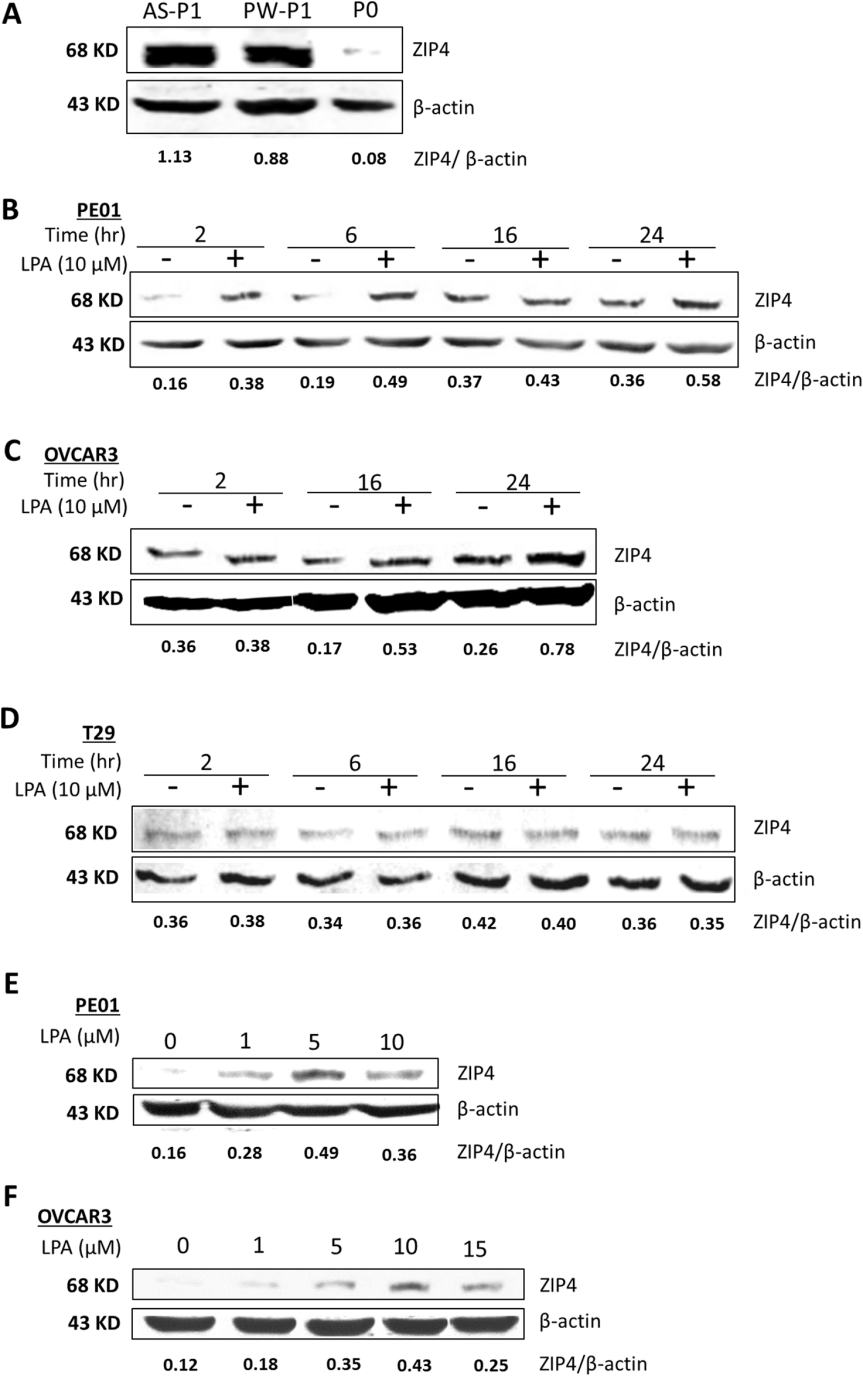
LPA-dose and -time dependently up-regulated ZIP4 via PPARγ in EOC cells **(A)** ZIP4 expression in ID8-P0 and ID8-P1 cells from ascites (AS-P1) and peritoneal wall (PW-P1). The relative expression levels were quantified using the ratios of ZIP4/β-actin (detected simultaneously in same blots using two colored fluorescence detection by the Li-Cor system as detailed in Supplementary materials) as shown in the figure. Five different ID8-P1 cell types (from different organs) were tested and all of them had elevated ZIP4. Only representative data are shown. **(B-D)** PE01, OVCAR3 and T29 cells were starved from serum for 16 hr prior to LPA (10 μM) treatment at different times as indicated. **(E-F)** PE01 and OVCAR3 were starved from serum for 16 hr prior to LPA treatment for different concentrations as indicated for 6 hr for PE01 and 16 hr for OVCAR3. The optimal LPA concentration and times to induce ZIP4 expression were 5-10 μM LPA and 2-6 hr for PE01 cells and 10 μM LPA and 16-24 hr for OVCAR3 cells. Representative results from ≥ three repetitive experiments are shown.

*Zip4* was shown to be 183-fold up-regulated in ID8-P1 vs. ID8-P0 cells in RNAseq data (Table 1). Here we confirmed ZIP4 overexpression in different ID8-P1 vs. ID8-P0 cells at the protein level (Figure 1A). Previously ZIP4 regulation had been exclusively studied in the context of Zn [7, 36]. The dramatic changes in ID8-P1 were only seen after cells were *in vivo* passaged, but not *in vitro*, strongly suggesting that the tumor microenvironment is important for these changes. We and others have shown that LPA is highly elevated in human EOC ascites [37], which represents an important part of the EOC tumor microenvironment. LPA can be produced by either EOC tumor or stromal cells [38, 39]. Thus, we tested whether LPA could regulate ZIP4 expression. LPA up-regulated ZIP4 in ID8 and human HGSOC cells (PE01and OVCAR3), but not in a human ovarian surface epithelial (HOSE) cell line T29 in a time- and dose-dependent manner (Figures 1B - 1F).

**Figure 2:**
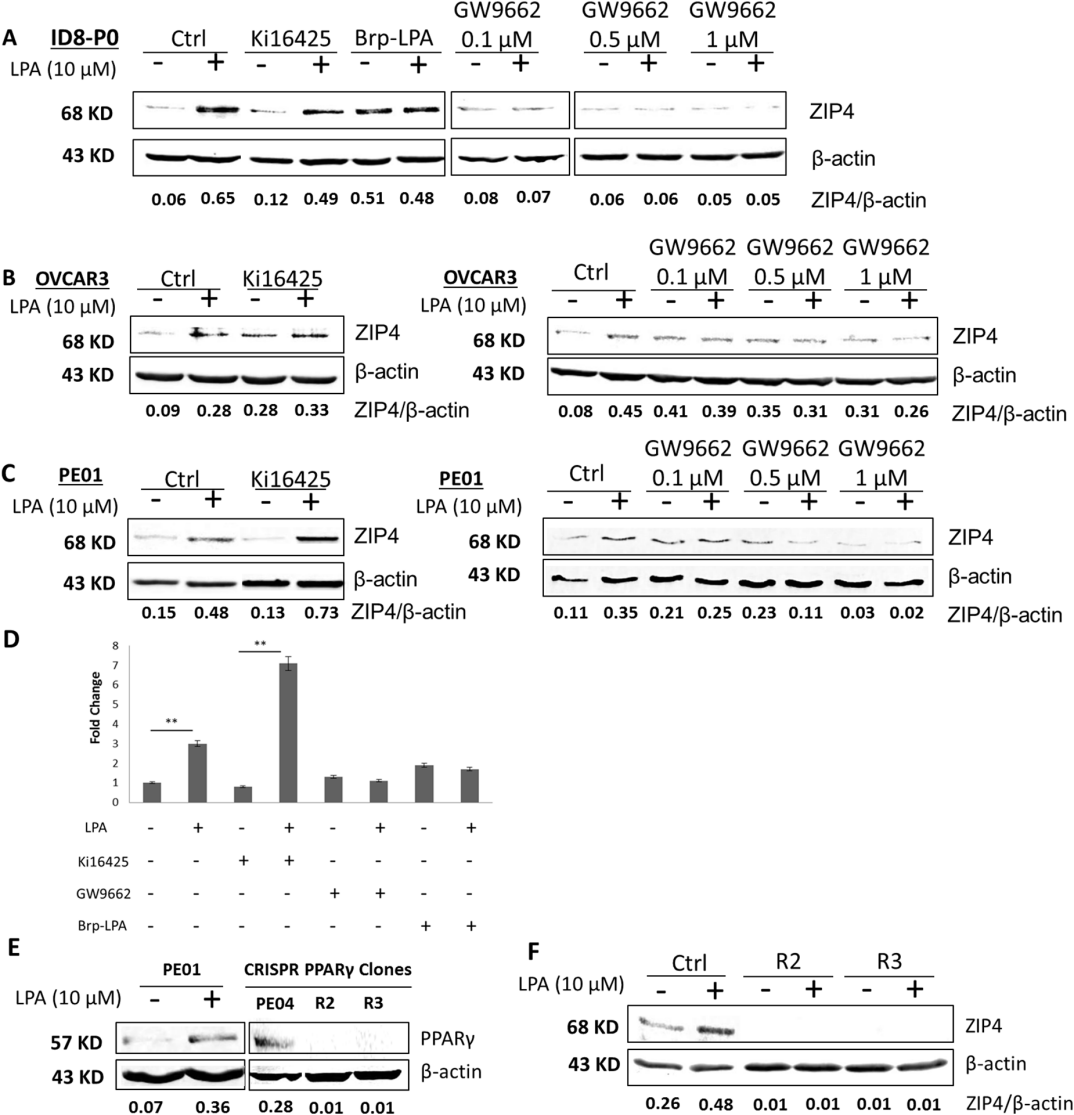
PPARγ was important for LPA induced ZIP4 up-regulation via in EOC cells **(A-C)** ID8, OVCAR3 and PE01 cells were starved from serum for 16 hr when the confluence was at ~80%. The cells were pre-treated with Ki16425 (10 μM), GW9662 (0.1 to 1 μM), or BrP-LPA (10 μM prior to stimulation with LPA (10 μM) **(D)** Summery of three independent experiments conducted in PE01 cells. **(E-F)** Human PE01 cell expressed lower level of PPARγ, which was up-regulated by LPA (10 μM). In PE04 cells, control-vector-transfected and CRISPR PPARγ-KO single clones were seeded into 24-well plate (2×10^5^ cells/ well) and cultured for 24 hr. Western-blot analyses were performed to examine the expression levels of PPARγ and ZIP4 in the presence or absence of LPA (10 μM) in PE01 and PE04 cells. Representative results from ≥ three repetitive experiments are shown.

The majority of LPA's known cellular effects are mediated by membrane GPCRs (LPAR_1-6_) [40]. In ID8-P0 cells, a LPAR_1_/LPAR_3_ selective inhibitor Ki16425 did not inhibit LPA-induced ZIP4 expression. BrP-LPA, a dual inhibitor of pan-LPA receptor and autotaxin (ATX [41]) activity, increased the basal level of ZIP4 and blocked the increased ZIP4 expression induced by LPA, assessed by fold of increase (Figure 2A). Similar trend for Ki16425's effect was observed in human HGSOC OVCAR3 and PE01 (Figures 2B - 2D). Figure 2D summarizes the fold changes observed in three independent experiments.

Different cell lines were used to show that the LPA-induced ZIP4 expression was not limited to one cell line. Importantly, HGSOC cells were used in the majority of experiments described below to demonstrate the clinical relevance of the studies. Although certain cell line variations exist, which are common scientific observations, our results suggesting that LPAR_1_ and LPAR_3_ are unlikely to be involved in LPA-induced ZIP4 expression.

On the other hand, the PPARγ selective inhibitor GW9662 dose-dependently blocked the effect (Figures 2A-2C), suggesting that LPA-induced ZIP4 was mainly mediated by PPARγ, and its GPCR receptors may be only partially involved. We used GW9662 under the conditions (concentration and treatment time) that major cell death was not apparent, evidenced and adjusted by actin loading and cell morphological observation. The inhibitor may also affect the basal level of ZIP4, but we focused on LPA-induced effect in this work. The Western analyses results from three independent experiments in PE01 cells are summarized in Figure 2D.

Compared to PE04 cells, PE01 cells expressed lower, but detectable levels of PPARγ, which was up-regulated by LPA (Figure 2E). GW9662 may have other actions and may have off-targets. PPARγ has a broad range of biological activities and is regulated by complex mechanisms [41]. In this work, we focused only on whether it was involved in LPA-induced ZIP4 up-regulation. To confirm the involvement of PPARγ in LPA-ZIP4 induction, we generated PPARγ-KO clones in PE04 cells using the Cas9 nuclease to facilitate RNA-guided site-specific DNA cleavage (CRISPR) system [42] (Figure 2E). As shown in Figure 2F, lack of PPARγ expression completely blocked ZIP4 expression in these cells, suggesting that PPARγ is necessary for ZIP4 expression. LPA-induced ZIP4 expression was sensitive to both the transcription and translational inhibitors actinomycin D (ActD) and cyclohexylamine (CHX) in mouse and human EOC cells (Supplementary Figures 3A, 3B). In addition, we conducted quantitative PCR (Q-PCR) analyses and found that the time course of mRNA expression induced by LPA (Supplementary Figures 3C, 3D) matched well to the protein levels in OVCAR3 and in PE01 cells (Figure 1), suggesting that ZIP4 was transcriptionally and translationally regulated.

### ZIP4 was functionally involved in proliferation, anoikis-resistance, and colony-formation in EOC cells

**Figure 3:**
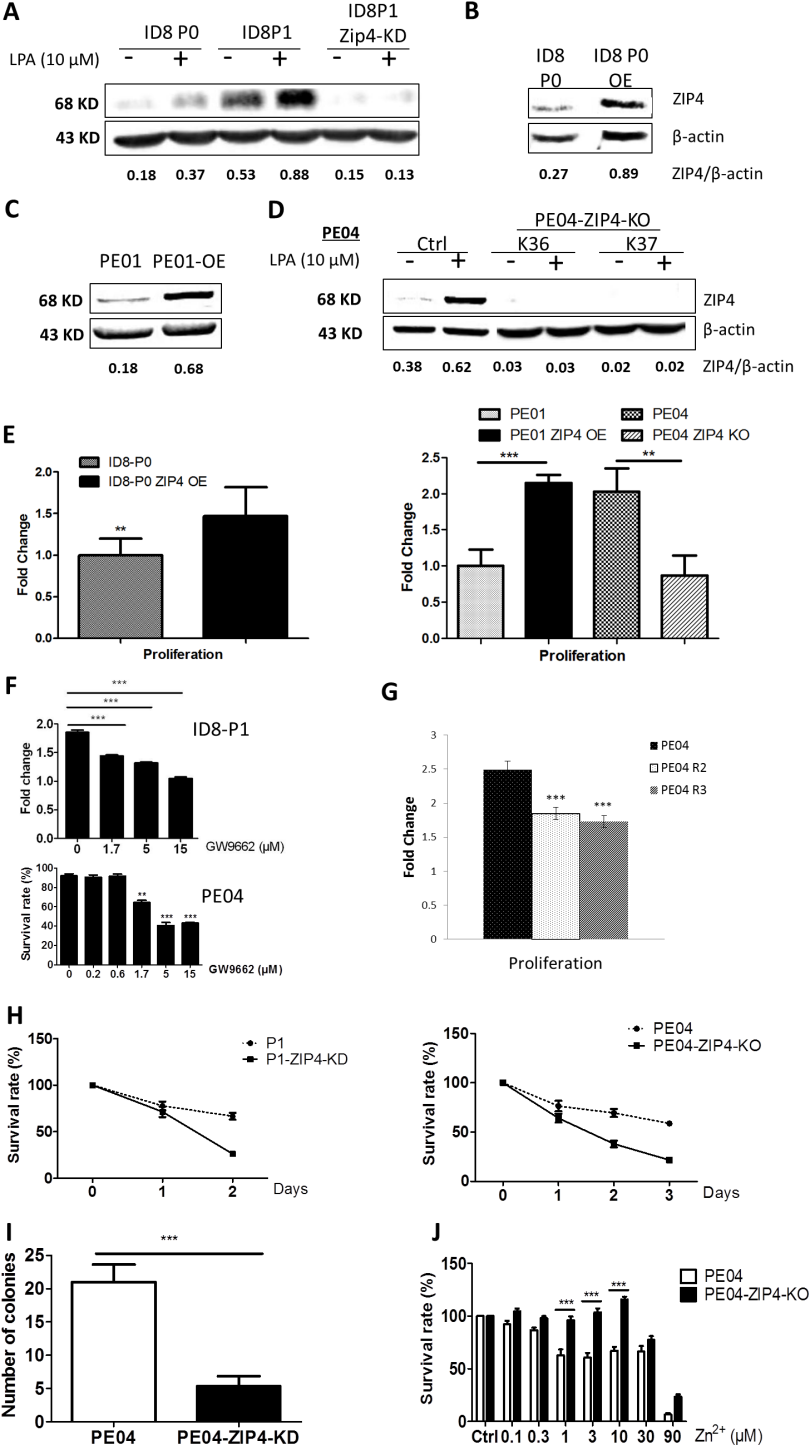
ZIP4 was functionally involved in cell proliferation, anoikis-resistance, and colony formation in HGSOC cells **(A)** LPA induced ZIP4 in ID8-P0 and ID8–P1 cells; ID8-P1-*Zip4*-KD clone was established using shRNA against *Zip4*. **(B)** ID8-P0 ZIP4 over-expression (OE) cells. **(C)** PE01-ZIP4-OE cells. **(D)** *ZIP4*-KO clones (K36 and K37) in PE04 cells. **(E)** ZIP4-OE in either ID8 or PE01 cells increased cell proliferation (48 hr to 72 hr) analyses using the MTT assays; conversely. ZIP4-KO in PE04 cells reduced cell proliferation. The proliferation was presented as fold changes normalized to vector-transfected PE01 cells (as one fold). **(F)** GW9662 dose-dependently inhibited cell proliferation in ID8-P1 and PE04 cells. **(G)** PE04-PPARγ-KO clones (R2 and R3) reduced cell proliferation. **(H)** Z*IP4*-KD in ID8-P1 cells and –KO in PE04 cells reduced anoikis-resistance. **(I)** PE04-Z*IP4*–KO cellspresented a reduced colony-formation. **(J)** Extracellular Zn induced cell death in PE04 cells; *ZIP4*-KO reduced Zn toxicity in the 1-10 μM range. Representative results from ≥ three repetitive experiments are shown. ^*^P < 0.05; ^**^P < 0.01; and ^***^P < 0.001.

To investigate the functions of ZIP4 in EOC, we generated ZIP4 knockdown (KD) or overexpression (OE) clones using shRNA against Zip4 in ID8 cells (Figures 3A – 3B). We also established human PE04-ZIP4-knockout (KO) clones using the CRISPR system and ZIP4-OE clones in human PE01 cells (Figures 3C – 3D). *ZIP4-*KD or -KO reduced and ZIP4-OE increased cell proliferation in both mouse and human EOC cells (Figure 3E). In addition, both ID8-P1 and PE04 cells were sensitive to the PPARγ inhibitor GW9962 in cell proliferation (Figure 3F). Moreover, cell proliferation was reduced in PPARγ-KO clones (Figure 3G). ZIP4-KD or -KO also significantly reduced anoikis-resistance (Figure 3H) and colony formation (Figure 3I) activities in EOC cells, supporting the importance of ZIP4 in EOC tumor promoting activities.

Most, if not all, of ZIP4's cellular functions have been linked to its ability to transport Zn, with its other Zn-independent mechanisms essentially unknown. To address the paradoxical observation that EOC tissues had high ZIP4 expression, but inhibitory effects of Zn in EOC cells, we tested whether ZIP4 was functional in transporting extracellular Zn in EOC cells. Consistent to published data, extracellular Zn was toxic to EOC cells (Figure 3J). ZIP4-KO in PE04 cells reversed Zn toxicity in the range of 1 to 10 μM, suggesting that ZIP4 is functional in extracellular Zn transporting. However, all of our LPA-related experiments were conducted in the absence of serum and extracellular Zn. Thus, we detected novel extracellular Zn-independent functions and/or regulations of ZIP4 in EOC cells.

### LPA and ZIP4 were involved in SP in EOC

**Figure 4:**
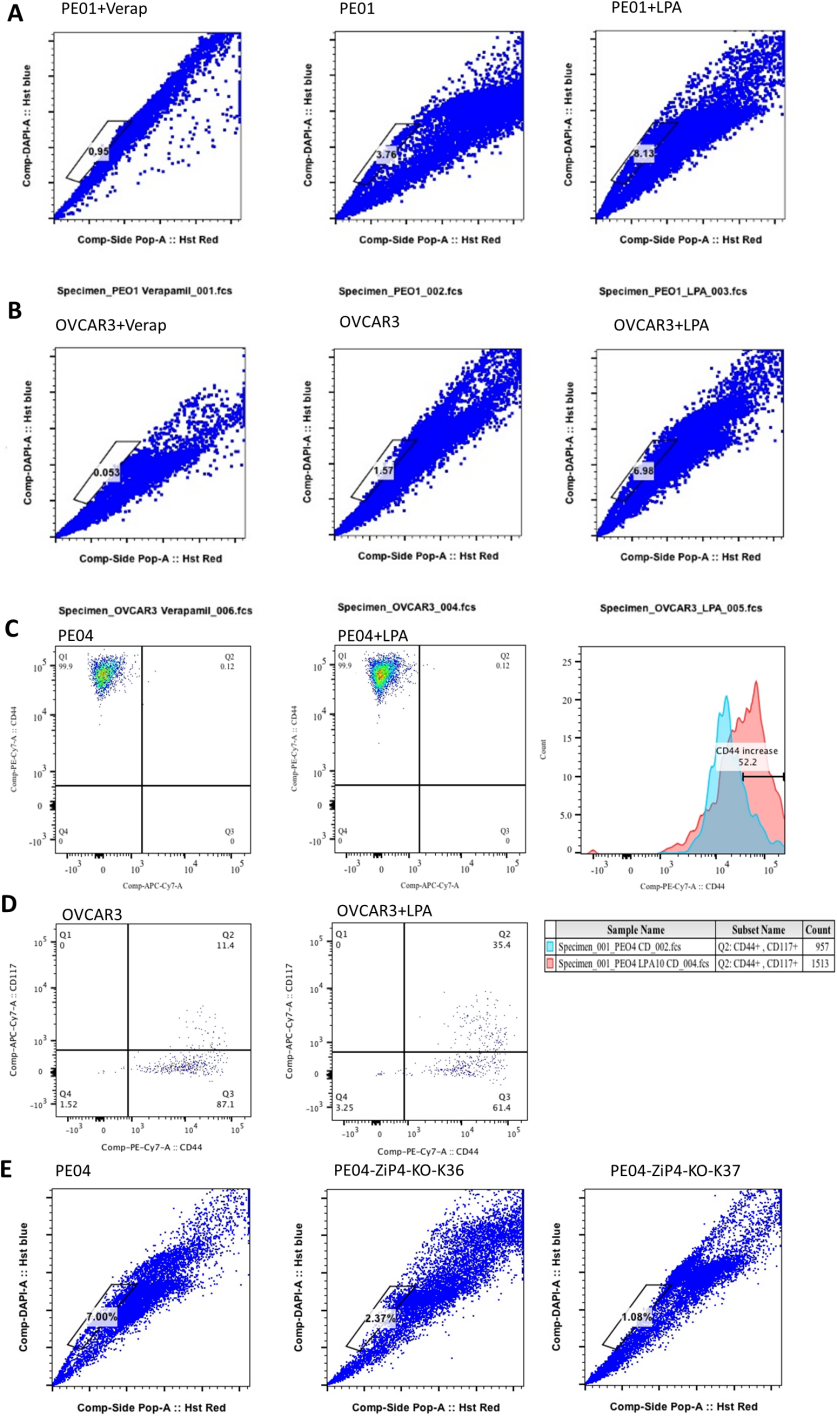
LPA and ZIP4 enhanced SP in HGSOC cells **(A-B)** Under the conditions that ZIP4 expression was induced in PE01 and OVCAR3 cells, LPA treatment increased SP % ≥ 2 fold. **(C)** > 99% of PE04 SP cells were CD44 positive; LPA did not alter the percent of CD44^+^ cells, but increased cell surface expression in SP cells. **(D)** LPA increased the percentage of CD44+CD117+ cells in OVCAR3. Cells were starved in all LPA related experiments. **(E)** ZIP4-KO dramatically reduced SP % in PE04 cells. These cells were not starved from FBS when the FACS analyses were done. Representative results from ≥ three repetitive experiments are shown.

Various CSC markers for EOC have been identified and the side-population (SP) cells are one of the most consistent markers for EOC CSC [16]. Using the Hoechst 33342 SP assays, we found that human EOC PE01 and OVCAR3 cells had ~ 3.8 and 1.6% cells in SP, respectively, which was inhibited by Verapamil (an inhibitor for drug efflux pump proteins and DNA-binding fluorophores that blocks SP), confirming that this is a real SP population. This SP population was increased to ~8.1% by LPA in PE01 cells (Figure 4A). LPA also increased the percent of SP in OVCAR3 cells by > 3-fold (Figure 4B).

Greater than 99% of SP in PE04 cells was CD44^+^ (a known EOC CSC marker) (Figure 4C). LPA shifted these cells to higher CD44 surface expression, without changing overall CD44 expression detected by cell staining (Figure 4C). In PE01 cells, CD44^+^CD117^+^ cells were low (~ 0.12%), which was increased by LPA to ~ 0.87% in SP population (Supplementary Figure 4). In OVCAR3 cells, CD44^+^CD117^+^ cells were ~11.4%, which was increased by LPA to ~ 35.4% in SP population (Figure 4D). Intriguingly, in the two ZIP4-KO clones (K36 and K37 in Figure 4E), the percent of SP reduced from 8.1 to 2.37 and 1.08% in PE04 cells, respectively, supporting the important roles of ZIP4 in SP and CSC.

### ZIP4 was involved in drug-resistance and spheroid-formation in EOC

**Figure 5:**
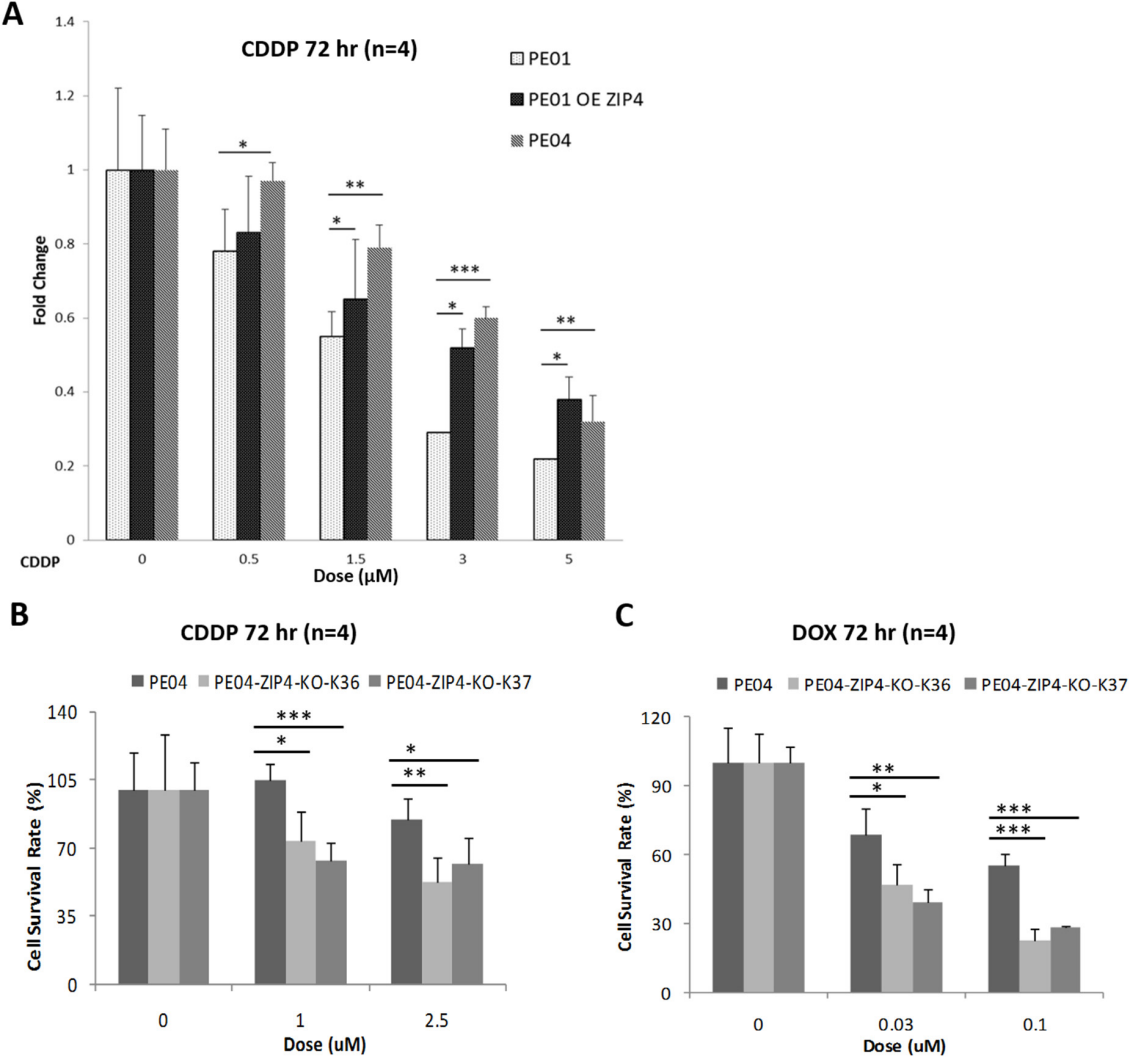
ZIP4 was involved in drug-resistance in HGSOC cells **(A)** PE01-ZIP4-OE increased resistance to CDDP-induced cell death. **(B-C)** PE04-ZIP4-KO increased cell sensitivity to CDDP- and Dox-induced cell death.

Cis-platinum (CDDP) or other platinum compounds are the most commonly used chemo-reagents for EOC. We tested the potential roles of ZIP4 in drug-resistance. PE01 and PE04 cell lines are from the same patient before (PE01) and after (PE04) the onset of multidrug resistance to CDDP, chlorambucil, and 5-fluorouracil [43]. As expected, *in vitro* assays showed that PE04 was more resistant to CDDP than PE01 cells (Figure 5A). Over-expression of ZIP4 PE01 cells increased resistance to CDDP and ZIP4-KO in PE04 cells reduced cell survival in the presence of CDDP, supporting the role of ZIP4 in DR, which has not been shown previously in any cells (Figures 5A and 5B). Similarly, ZIP4 was also involved in drug-resistance to DOX (Figure 5C).

**Figure 6:**
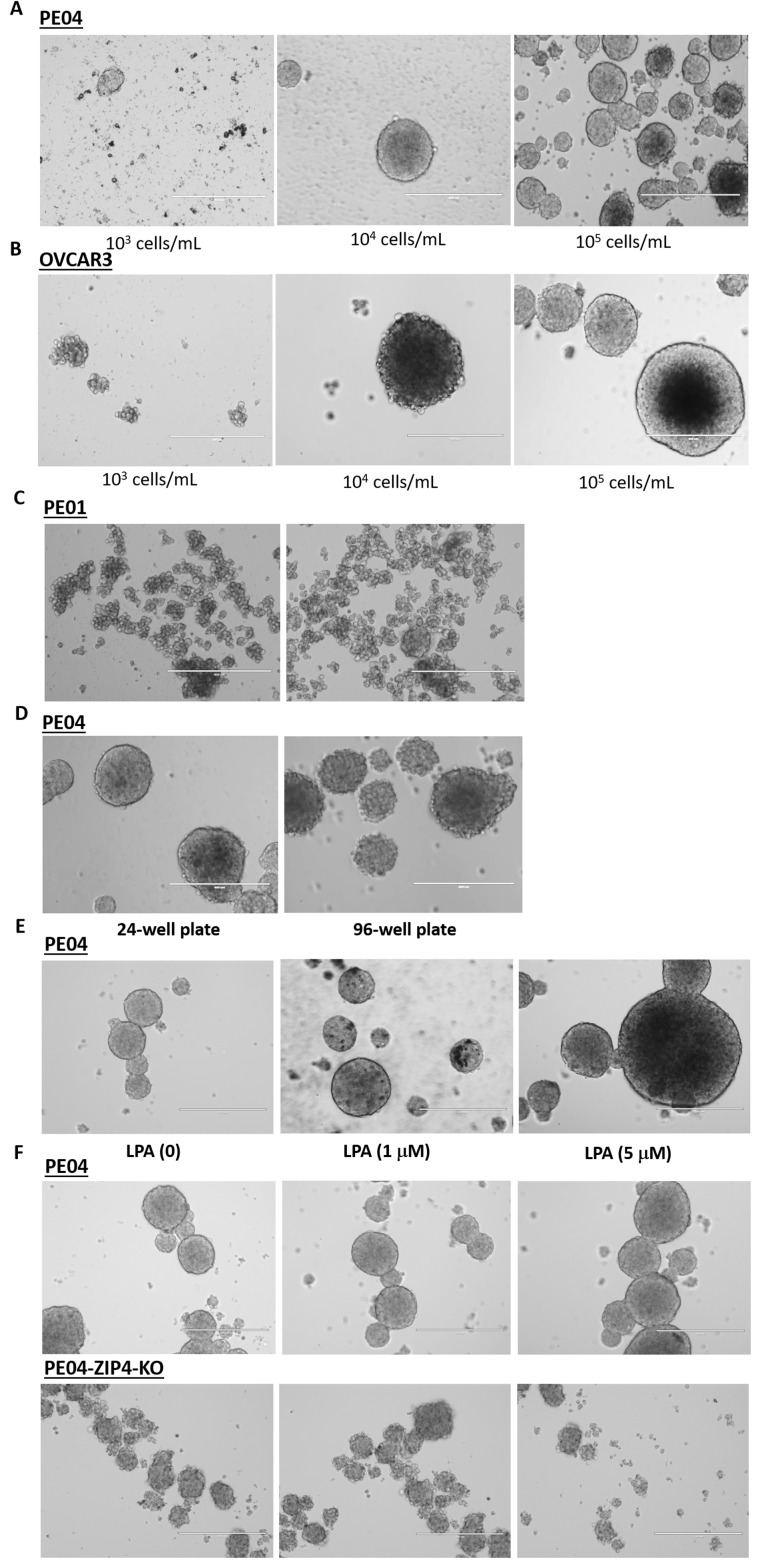
LPA and ZIP4 stimulated spheroid-formation in HGSOC cells **(A-B)** PE04 and OVCAR3 cells were cultured in ultra-low-attachment plates; spheroid-formation was cell density dependent. Cells were cultured in stem cell conditions (see Methods for details) for 7 days. **(C-D)** Under the same conditions (1×10^4^/mL in either 24- or 96-well plates), PE04 formed larger or more complete spheroids than PE01. **(E)** PE04 cells formed more complete spheroids under LPA treatment. **(F)** ZIP4-KO cells formed fewer and smaller spheroids when compared to control cells. The scale bars in D to I = 400 nm. Representative results from ≥ three repetitive experiments are shown.

Spheroids, in general, have high SP, drug-resistance, and CSC activity [20]. Spheroids are present in the malignant ascites of essentially all EOC patients and represent a significant impediment to efficacious treatment due to their roles in progression, metastasis, and drug-resistance [44]. LPA has been shown recently to be potent spheroid inducer in EOC cells [33]. PE01, PE04, and OVCAR3 were all able to form spheroids using the stem cell culture conditions in suspension [19, 33]. The spheroid- formation was dependent on the cell density used (Figures 6A to 6B). Under the same conditions, PE04 cells formed more and/or larger spheroids than PE01 (Figures 6C and 6D). LPA stimulated bigger spheroid formation in PE04 cells (Figure 6E).

We tested spheroid-formation in different ZIP4-KO clones. ZIP4-KO had dramatic inhibitory effects on spheroid-formation. In Figure 6F, the top three panels show the representative spheroids formed in different fields in PE04 cells. Using the same conditions (10^4^/mL), ZIP4-KO clones did not have the capacities to form spheroids (the bottom 3 panels).

### ZIP4 was involved in tumorigenesis and CSC activities *in vivo*

**Figure 7:**
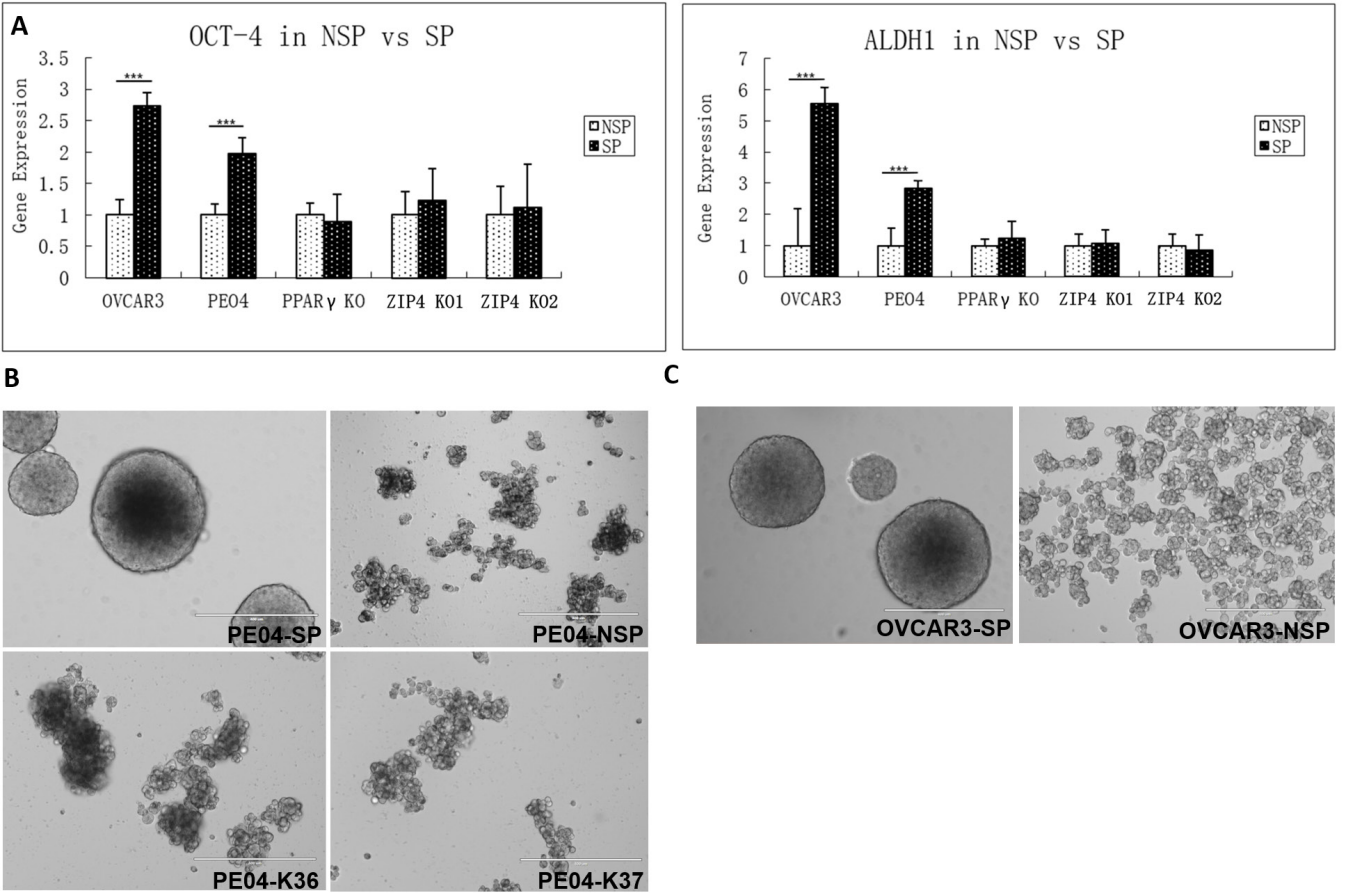
ZIP4 increased CSC properties **(A)**. The two known EOC CSC markers, ALDH1 and OCT4 expressed at significantly higher levels in both OVCAR3 and PE04 SP vs. non-SP cells. ZIP4-KO in PE04 cells completely suppressed the high expression of these two genes in SP cells. **(B)** Only SP cells formed spheroids. When ZIP4 was KO in PE04 cells, even though SP cells could still be isolated using Hoechst-exclusion FACS, but these cells could not form spheroids. The SP portion and non-SP portion were separated by BD SORP FACSAria (BD Biosciences, San Jose, CA) after Hoechst-33342 Staining. 1×10^4^ cells per well into Corning 24 Well Plate with low attachment surface under stem cell culture conditions. After 7 days in culture, the SP portion generated spheroids from the PE04/OVCAR3 primary cells, but the non-SP portion cannot form any spheroid. **(C)** Similar results in spheroid-formation were observed in OVCAR3 SP and non-SP cells.

To further characterize the role of ZIP4 in SP cells, we separated SP and non-SP (FACS-sorted) in PE04 and OVCAR3 cells. Only SP cells formed spheroids and up-regulation of known EOC stem cell markers Oct4 and ALDH1 (Figures 7A - 7C).

**Figure 8:**
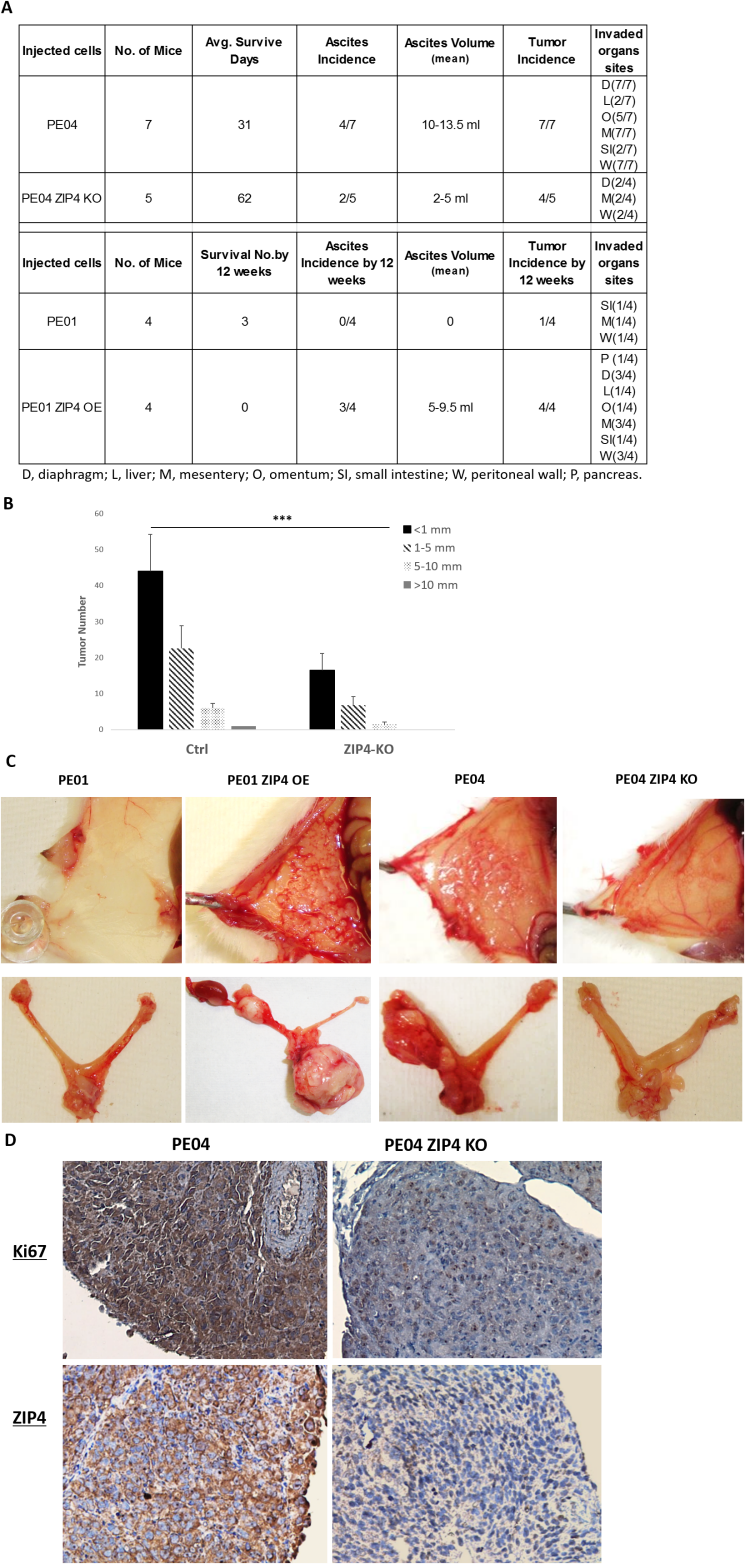
ZIP4 was positively involved in tumorigenesis *in vivo* **(A-B)** Summary of tumor and ascites development of the control (PE01 and PE04), PE01-ZIP4-OE, and PE04-ZIP4-KO cells injected into NSG mice (n=4-7 in each group). **(C)** Representative tumor pictures of tumors on the peritoneal wall and the ovary. **(D)** Representative immunostaining of ZIP4 and Ki67 (a proliferation marker) in the tumor sections derived from wild type and ZIP4 KO PE04 cells.

The i.p. injected PE04 control cells (vector-transfected) developed tumors and ascites in 4-5 weeks in mice, with an average survival day of 31 (n=7, Figure 8A). ZIP4-KO (combined K36 and K37 ZIP4-KO cells; mice n=5) dramatically reduced tumorigenesis and these mice had an average survival day of 62. Although tumors developed in each of the four ZIP4-KO cell injected mice, 2 of 5 developed ascites, compared to 4 of 7 in the control group. In addition, tumor numbers and sizes, as well as metastatic sizes were significantly reduced in ZIP4 KO cell injected mice (Figures 8A – 8B). PE01 cells developed tumors much slower than PE04 cells. By 12 weeks, only 1 of 4 mice with i.p. injected PE01 cells (5 × 10^6^) had developed tumors. In contrast, 4 of 4 mice with i.p. injected PE01-ZIP4-OE cells (5 × 10^6^) had developed tumors and/or ascites (Figure 8A). Representative tumor nodules grew in the peritoneal walls and/or in the ovaries from WT, ZIP4-KO, ZIP4-OE cell injected mice are shown in Figure 8C.

Injection of only 500-5000 SP formed tumors in 8 to 12 weeks (n=5 in total). In contrast, injection of up to 5,000 non-SP cells did not formed tumors in 3 months (n=4), supporting the CSC properties of SP cells in EOC. Lack of ZIP4 expression in tumors derived from ZIP4 KO cells was confirmed by IHC and tumors derived from wild-type PE04 cells had higher levels of proliferation as detected by the proliferative marker Ki67 (Figure 8D).

## DISCUSSION

Our data presented here confirmed ZIP4 over-expression in EOC tissues (Supplementary Figures 1-2) and support an innovative hypothesis that the apparent disconnection between Zn and ZIP4's effects in EOC (as mentioned in the Introduction) is due to, at least in part, the fact that ZIP4 is able to mediate LPA's tumor promoting activity in an extracellular Zn-independent manner. The novel regulation and signaling of the LPA-ZIP4 axis provides paradigm-shifting concepts for studies in ZIP4 and possibly other solute transporting proteins. ZIP4 may represent as a prototype in this large family of proteins, for their solute transporting-independent signaling and regulatory mechanisms. The molecular mechanisms by which ZIP4 exerts its extracellular Zn-independent signaling remain to be investigated.

This is the first report of ZIP4's role in EOC. ZIP4 stimulates cell proliferation, anoikis-resistance, stem cell-related cellular activities (such as SP, spheroid formation, and CSC marker expression), and drug-resistance *in vitro* and tumor progression *in vivo*. In particular, the original choice of ZIP4 is from one of top candidate genes in highly aggressive and quickly progressed EOC cells vs. less aggressive EOC cells. Whether ZIP4 is involved in EOC grade, metastasis, recurrence, survival, and/or progression free survival in human tumors will be a highly interesting area to be further explored. Nevertheless, many genes/proteins have proven to be important cancer targets, even though they may not be involved in all steps of cancer progression.

Our *in vitro* and *in vivo* data provide the first lines of evidence that ZIP4 is likely to play crucial promoting roles in drug-resistance, SP, and CSC of HGSOC. In particular, ZIP4 strongly influences SP, spheroid formation, and *in vivo* tumorigenesis in HGSOC, which is consistent with its high expression in EOC tissues. These data suggest that *ZIP4* is a novel target for EOC, with the LPA-PPARγ axis as one of its important up-stream regulators.

Seo *et al* have shown that autotaxin (ATX, the major LPA-producing enzyme) stimulates maintenance of EOC CSC through LPA- and LPAR_1_-mediated autocrine mechanisms in EOC cells [33]. While our data are highly consistent with LPA's roles in EOC CSC as reported, the LPA-PPARγ-ZIP4 signaling pathway is novel, which has not been shown in any other cells. The majority of the data generated in Seo's work used A2780 and SKOV3 cell lines, which are not HGSOC [45, 46]. It is possible that the LPA-PPARγ-ZIP4 pathway is cell line-dependent and/or that LPAR- and PPARγ-mediated LPA effects co-exist in EOC cells.

The majority of published LPA biological effects are mediated by its plasma membrane LPAR_1-6_. All current LPA blocking strategies and clinical trials related to cancer focus on its membrane receptors (LPAR_1-6_) and on LPAR_1-3_ more particularly [29]. At least three compounds blocking these receptors have passed phase I and phase II clinical trials [29]. While our Ki16425 data suggest that LPAR_1_ and LPAR_3_ are unlikely to be involved in LPA-induced ZIP4 up-regulation, the results from BrP-LPA suggest that LPAR_2,4-6_ and/or ATX may be partially involved in non-LPA related basal level ZIP4 regulation, which may be related to BrP-LPA's off-targeting activities. This needs to be addressed using other approaches including genetic manipulations. Nevertheless, the current study is focused on the clear involvement of PPAR*γ* in the LPA induced effect. Although LPA has been identified as a ligand for PPAR*γ* [31, 47], the LPA-PPAR*γ* studies are mainly limited to the vascular and metabolic processes [32, 48]. The roles of PPAR*γ*-mediated LPA effects in cancer are essentially unknown.

PPAR*γ* is a nuclear hormone receptor that mediates the effects of fatty acids and their derivatives at the transcriptional level [41]. HGSOC patients with a high expression level of PPAR*γ* had significantly poorer overall survival [49]. Acyl-LPAs (only those with unsaturated fatty acid) bind PPAR*γ* with affinities similar to that of the synthetic full agonist of PPAR*γ*, rosiglitazone (Rosi), but their binding sites are different [49]. Interestingly, the biological effects of PPAR*γ* may be ligand, cell type, and context-dependent. LPA and Rosi may induce similar or opposing effects in a cell context-dependent manner [32, 50]. In addition, activating PPAR*γ* by its other ligands appears to possess both pro- and anti-cancer activities. Even in the same cancer, such as colon and EOC, opposing results have been reported [32, 51–53]. The mechanisms underneath these paradoxical observations warrant additional investigation. Nevertheless, while our results do not completely rule out LPAR_1-6_-dependent actions, ZIP4's regulation by LPA via PPARγ is a new finding, which suggest that co-targeting both groups of LPA receptors (GPCRs and PPAR*γ*) is critical.

PPAR*γ* plays an important role in stroke, cardiovascular, age-related macular degeneration, and other inflammation-related diseases. PPAR*γ* agonists (not antagonists or inhibitors) confer benefits in diabetes and atherosclerosis, two known risk factors associated with cardiovascular disease. However, deleterious effects have limited their clinical usages [54–56]. Since chronic inflammation is a well-known factor closely associated with cancer [57, 58], and PPARγ activation by synthetic agonists will preferentially bind with retinoid X receptor α and signal antiproliferative, antiangiogenic, and prodifferentiation pathways in several tissue types, these agonists have been tested for their anti-cancer effects. Thiazolidinediones (TZDs) are PPARγ agonists and orally effective medicines for metabolic syndrome and type 2 diabetes. Although data from human trials suggest the efficacy of TZDs as monotherapy in prostate cancer and glioma and as chemopreventive agents in colon, lung, and breast cancer, the action of TZDs are highly complex and those actions do not correlate with cellular PPARγ expression status and/or activation [59, 60]. Our results also imply that different agonist types may induce distinct effects, suggesting these issues require significant additional investigation before successful PPARγ targeting in clinical settings.

The regulation and activities of ZIP4, an important Zn transporter, have been exclusively studied in the context of Zn [6, 36, 61]. The *ZIP4* promoter has only been minimally studied in mouse brain tissue and mouse intestinal epithelial cells [62]. Initial search in the 2.3 kb ZIP4 promoter region did not identify a classical PPAR*γ*-cis element. This suggests either longer promoter regions or other elements need to be examined, or an indirect PPAR*γ* effect is involved.

Our preliminary data suggest that PE01 (or PE04) and OVCAR3 cells may have differential epigenetic regulations [e.g. trichostatin A, an inhibitor of histone deacetylase (HDAC) families of enzymes, did not affect LPA-induced ZIP4 in PE01 cells but blocked this action in OVCAR3 cells; our unpublished observations). These additional layers of regulation may affect the optimal time of LPA-induced ZIP4. In addition, we found that ZIP4 expression could be stress stimulated. Cell starvation appears to have regulatory effect on ZIP4, and hence the basal levels of ZIP4 during starvation may change as we observed in Figure 1. Moreover, ZIP4 appeared to be doublets in ID8 cells in Western blots detected by a mouse anti-ZIP4 antibody, but the human ZIP4 antibody (cross-react with mouse ZIP4) detected ZIP4 as a single band. These aspects warrant further investigation.

Taken together, we have revealed an innovative LPA-PPAR-ZIP4 signaling pathway and provide strong data to support ZIP4's tumor promoting activities in EOC, and in human HGSOC cells in particular. Importantly, ZIP4 plays pivotal promoting roles in EOC CSC, which are the critical target for EOC treatment.

## MATERIALS AND METHODS

### Reagents, cell lines and culture

Oleoyl-LPA was from Avanti Polar Lipids (Birmingham, AL). The following reagents were used: Ki16425 (Calbiochem, San Diego, CA); BrP-LPA (EBI, Salt Lake City, UT); C3 exoenzyme (Cytoskeleton, Denver, CO); GW9662 (EMD Corp; Billerica, MA); and actinomycin D (ActD) and cyclohexamide (CHX; Sigma-Aldrich, St. Louis, MO). Alexa fluor-conjugated secondary antibodies were from Life Technologies (Grand Island, NY). Mouse ZIP4 antibody (AF7315) was from R&D Systems (Minneapolis, MN). Human ZIP4 (20625-I-AP) and PPARγ (H100) antibodies were from Proteintech (Rosemont, IL) and Santa Cruz (Paso Robles, CA). Mouse ZIP4 antibody was from Santa Cruz (Paso Robles, CA). The pair of PE01/PE04 cell lines was from Dr. Daniela Matei (Northwestern University); the OVCAR3 cells were obtained from ATCC (Manassas, VA). The ID8, T29, and OVCA433 cell lines were kind gifts from Dr. R. Bast (M.D Anderson), Dr. Jinsong Liu (M.D Anderson), and Dr. Paul F Terranova (University of Kansas Medical Center), respectively. These cell lines were authenticated by ATCC. ID8-P1 cells were obtained as we published previously [2]. In brief, ID8-P0 cells (5×10^6^) were injected into the peritoneal cavity of C57-BL6 mice. Between 80 and 85 days post injection, tumor nodules on the peritoneal wall were isolated and cultured with Zeocin (100 μg/mL) to select tumor cells. The new cell lines were termed ID8-P1. All cell lines were maintained in a humidified atmosphere at 37°C with 5% CO2. OVCAR3 cells were maintained in RPMI-1640 supplemented with 20% FBS (ATCC, Manassas, VA), 0.01 mg/mL insulin and 50 U/mL penicillin, and 50 μg/mL streptomycin. PE01/PE04 cells were cultured in RPMI 1640 with glutamine, 10% FBS, and 100 μg/mL Penicillin-Streptomycin-Amphotericin B. For serum starvation, cells were incubated in the basal medium without FBS or antibiotics. LPA treatment was performed in cells starved from serum for 16-24 hr.

### RNA-sequencing analysis and stable clones

Stranded whole transcriptome RNA-seq was performed as we have described [35]. Briefly, biological duplicates of cells (10^7^) were lysed and RNA was extracted according to manufacturer's protocol (Qiagen RNeasy Mini kit). Total RNA was fractionated by size using ethanol concentration manipulations. The large RNA fraction (>200 nt) was fragmented prior to library construction. Ribosomal RNA was reduced by duplex specific nuclease (DSN) following limited hybridizations of both fractions and then amplified to add barcodes for multiplexing on the Illumina HiSeq2000 platform. Demultiplexing was performed by CASAVA v1.8.2 and trimming was accomplished with Trimmomatic v0.22 with additional trimming by fastx_clipper v0.0.13.2. Read mapping was performed by tophat2 v2.0.6 to the human genome hg19 (UCSC) with Gencode annotation v13 allowing no more than two mismatches. *ZIP4 and PPARr CRISPR* lentiVirus vectors (GeneCopoeia, Rockville, MD) were used for gene knockout transfected to 293T cells for virus packaging. PE01/PE04 and OVCAR3 cells were infected by virus 3 times and stable clones were selected by puromycin (0.5 μg/mL). More details of the methods are described in Supplementary Materials.

### Western blot analysis

Western blot analyses were conducted using standard procedures and proteins were detected using primary antibodies and fluorescent secondary antibodies (IRDye 800CW-conjugated or IRDye 680-conjugated anti-species IgG, Li-Cor Biosciences, Lincoln, NE) as we described previously [2]. The fluorescent signals were captured on an Odyssey Infrared Imaging System (Li-Cor Biosciences, Lincoln, NE) with both 700- and 800-nm channels. Boxes were manually placed around each band of interest, and the software returned near-infrared fluorescent values of raw intensity with background subtraction (Odyssey 3.0 analytical software, Li-Cor Biosciences, Lincoln, NE). The protein MW marker used was the Pre-stained SDS-PAGE Standards, broad range (BIO-RAD, Cat. Log # 161-0318).

### Cell proliferation, anoikis-resistance, and spheroid-formation assays

Cell (2×10^3^) were cultivated in tissue culture 96-well plates (TPP, Switzerland in Europe) in growth medium, cultured for 48 hr, 10 μl of the MTT dye solution (Sigma, USA) (5 mg/ml in phosphate buffer saline) was added to the cells; the cells were incubated at the same conditions for 3 hr. The supernatant was removed after centrifugation (1500 rpm, 5 min), 100 μl of dimethyl sulfoxide was added to each well to dissolve formazan. The absorption was measured by a Multilabel Counter (VICTOR3, Perkin Elmer, USA) at a wavelength of 540 nm. At least three independent experiments were conducted. In each experiment set, the same cell numbers were seeded for control and all testing cell lines with 3-8 wells/data point. The mean absorption values at 540 nm from the control cells were used as one fold in each figure and the proliferation results from testing cell lines were presented as fold changes of the absorption values when compared to that of the control cells. Anoikis-resistance and soft agar colony assays were described in detail previously [2]. Single cells were re-suspended at 1×10^3^ to 1×10^5^ cells/mL in serum-free DMEM/F12 supplemented with 5 μg/mL insulin (Sigma), 20 ng/mL human recombinant epidermal growth factor (EGF; Invitrogen), 10 ng/mL basic fibroblast growth factor (bFGF; Invitrogen), and 0.4% bovine serum albumin (BSA; Sigma), followed by culturing in 24- or 96-well Ultra Low Attachment plates (Corning, NY). Spheroids were photographed after seven days in culture.

### Quantitative real-time PCR

RNA was extracted with the RNeasy mini kit (Qiagen, Valencia, CA) and reverse transcribed by M-MLV reverse transcriptase. Quantitative real-time PCR was performed on a Light Cycler 480 (Roche, Indianapolis, IN) with a SYBR Green I Master Mix (Roche, Indianapolis, IN). mRNA abundance was normalized to GAPDH. Negative controls contained no reverse transcription or the reverse transcriptase. RNAs from triplicate cell pellets per condition were analyzed. Relative gene expression was calculated using the method given in Applied Biosystems User Bulletin No.2 (P/N 4303859B). Primer pairs used in this study were: GAPDH: F, 5’-CACCATTGGCAATGAGCGGTTC-3’/R, 5’-AGGTCTTTGCGGATGTCCACGT-3’; ZIP4: F, 5’-ATGTCAGGAGCGGGTCTTGC-3’/ R, 5’- GCTGCTGTGCTGCTGGAAC-3’. ALDH1: F, 5’- CTGCTGGCGACAATGGAGT-3’/R, 5’- GTCAGCCCAACCTGCACAG-3’; Oct4: F, 5’- TCAGGTTGGACTGGGCCTAGT-3’/R, 5’- GGAGGTTCCCTCTGAGTTGCTT-3’.

### FACS side-population (SP) analyses

Starved cells (5×10^5^) were treated with LPA under the ZIP4-induction conditions. Cells were detected by acutase and trypsin and re-suspended in DMEM^+^ (DMEM+10 μM HEPES + 2% FBS). Verapamil (10 μM, 37° C for 30 min) was used in control assays to ensure that the gated SP populations were indeed SP. Hoechst 3442 (5 μg/mL) was added and cells were incubated at 37° C for 90 min. After centrifugation at 2200 rpm for 5 min, cells were re-suspended in Cold HBSS^+^ buffer (Hanks’ Balanced Salt Solution +10 μM HEPES + 2% FBS; 10^6^ cells/ mL). Labeling CD44 and/or CD117 was conducted at 4° C for 30 min. Cells were re-suspended at 2×10^6^ cells/mL in ice cold HBSS+ and the FACS analyses were conducted in BD SORP FACSAria (BD Biosciences, San Jose, CA) for SP sorting and analysis and BD LSR Fortessa (4 laser) Analyser (BD Biosciences, San Jose, CA) for surface marker analysis (CD44/CD117).

### Human tissue immunohistochemistry

Normal ovary, benign ovary, and ovarian cancer tissues were purchased from the Cooperative Human Tissue Network (CHTN; Philadelphia, PA); the usage of these tissues was approved by an Indiana University School of Medicine IRB as described previously [34]. Standard IHC procedures were used. A tissue microarray containing ovarian high-grade serous carcinoma in triplicate 1.0-mm cores, along with other gynecological neoplasms and controls, was constructed using de-identified samples from patients whose cases were reviewed by the Johns Hopkins Department of Pathology. Construction was performed by the Oncology Tissue Services at Johns Hopkins University. Institutional Review Board approval was obtained for this study cohort.

### Xenograft mouse model

Female NSG mice were obtained from the *In Vivo* Therapeutics Core, Indiana University School of Medicine (Indianapolis, IN). At 7 to 10 weeks of age, PE01 or PE04 cells [Vector-transfected control, ZIP4-KO, ZIP4-overexpression (OE), or PPARγ-KO, 5×10^2^ to 5×10^6^ in 500 μL of PBS] were i.p. injected into mice. Tumors were monitored daily. Mice were euthanized after tumor and ascites development. Tumors were counted at each metastatic location, and tumor diameters were measured. Animal protocols were approved by the Indiana University School of Medicine Animal Care and Use Committee.

### Statistical analyses

The Student's t-test was utilized to assess the statistical significance of the difference between two treatments. The asterisk rating system as well as quoting the *P* value in this study was ^*^ *P* < 0.05; ^**^ *P* < 0.01; and ^***^ *P* < 0.001. A *P* value of less than 0.05 was considered significant.

## SUPPLEMENTARY MATERIALS FIGURES AND TABLES



## Abbreviations

ABC: ATP-binding cassette
ActD: actinomycin D
ATX: autotaxin
bFGF: basic fibroblast growth factor
BSA: bovine serum albumin
CHX: cyclohexamide
CSC: cancer stem cells
CA: constitutive active
CDDP: Cis-platinum
CHTN: the Cooperative Human Tissue Network
CRISPR: the Cas9 nuclease to facilitate RNA-guided site-specific DNA cleavage
Dox: doxorubicin
EOC: Epithelial ovarian cancer
FACS: fluorescence-activated cell sorting
GPCR: G protein-coupled receptors
HDAC: histone deacetylase
HGSOC: high-grade serous ovarian cancer
IHC: immunohistochemistry
LPA: lysophosphatidic acid
KD: knockdown
KO: knockout
LGSOC: low-grade serous ovarian cancer
LPA: lysophosphatidic acid
LPAR: LPA receptors
OE: over-expression
PPARγ: the nuclear receptor peroxisome proliferator-activated receptor gamma
pYAP: phosphorylation of YAP
Rosi: rosiglitazone
SP: side-population
YAP: Yes-associated protein
ZIP4: the zinc transporter 4; gene name Slc39a4
Zn: Zinc
