# Supplementary material for: The novel ZIP4 regulation and its role in ovarian cancer
Source: Oncotarget. 2017 Sep 30;8(52):90090–107. doi: 10.18632/oncotarget.21435 (PMC5685734; doi:10.18632/oncotarget.21435)
Supplement: Supplementary file 1 [file oncotarget-08-90090-s001.pdf]

The novel ZIP4 regulation and its role in ovarian cancer

SUPPLEMENTARY MATERIALS

ZIP4 was over-expressed in human EOC tissues

The TCGA and Oncomine data suggest that the *ZIP4* gene is over-expressed in EOC [14]. We confirmed the over-expression of ZIP4 in EOC using a subset of tissues obtained from CHTN [33]. ZIP4 protein was over-expressed in EOC vs. benign and normal ovarian tissues (Supplementary Figure 1; representative data). We also used an ovarian cancer TMA to evaluate ZIP4 expression. The results are summarized in Supplementary Table 1. Twelve (12) of 16 (75%) of HGSOC samples expressed high levels of ZIP4 (scored +++++ or ++++ in Supplementary Table 1). The remaining (4 of 16) HGSOC tissues also expressed ZIP4, albeit with lower levels. Only 1 of 4 (25%) LGSOC tissue samples expressed high level of ZIP4 and none of other groups of tissues (ovarian endometriod carcinoma, serous borderline ovarian cancer, and control tissues) expressed high levels of ZIP4. Representative results are shown in Supplementary Figure 2. The tissue from normal breast was used as a negative control and is not related to the breast cancer tissue.

LPA regulated ZIP4 transcriptionally

In both mouse ID8 cells (Supplementary Figure 3A) and human HGSOC cells (Supplementary Figure 3B), the transcription and translational inhibitors actinomycin D (ActD) and cyclohexylamine (CHX) inhibited LPA-induced ZIP4 in HGSOC cells. In addition, we conducted quantitative PCR (Q-PCR) analyses and found that the time course of mRNA expression induced by LPA (Supplementary Figure 3C, 3D) matched well to the protein levels in OVCAR3 and in PE01 cells (Figure 1, main text) suggesting that ZIP4 was transcriptionally and translationally regulated.

LPA increased CSC marker expression in HGSOC Cells

In PE01 cells, $CD44^{+}CD117^{+}$ cells were low (~ 0.12%), which was increased by LPA to ~ 0.87% in SP population (Supplementary Figure 4).

Supplementary Table 1: Summary of ZIP4 expression in TMA samples

Samples (number of subjects; N)	Scores (N)	Note
HGSOC (16)	+++++ (10)<br>++++ (2)<br>+++ (1)<br>++ (2)<br>+ (1)	Stage III or higher (13)<br>Stage IV (3)
LGSOC (4)	++++ (1)<br>+++ (1)<br>+ (1)<br>Neg (1)	
Ovarian endometrioid carcinoma (2)	+ (2)	Stage III (2)
Serous borderline ovarian cancer (1)	Only surrounding-edge areas were positively stained (1)	Stage III (1)
Fallopian tube carcinoma (1)	Neg (1)	Stage II (1)
Breast control tissue (1)	Neg (1)	

+ to +++++, degree of positive staining, with +++++ as the strongest; N, number of subject; Neg, negative; HGSOC, high-grade serous ovarian cancer; LGSOC, low-grade serous ovarian cancer.

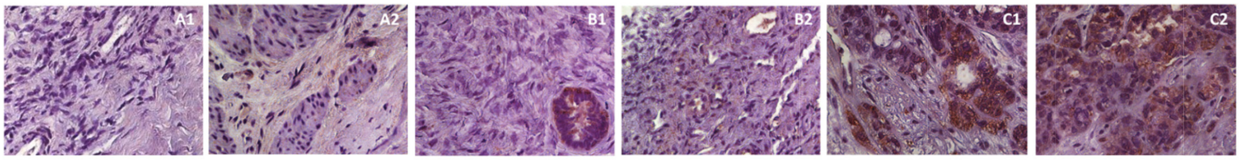

Supplementary Figure 1: ZIP4 expression in human ovarian tissues. **(A)** Normal ovarian tissues; **(B)** benign gynecological tumors; **(C)** EOC tissues. Samples used: normal, n=5; benign, n=4; and EOC, n=5. Consistent results were obtained for different groups of samples. Two representative panels from each group are shown.

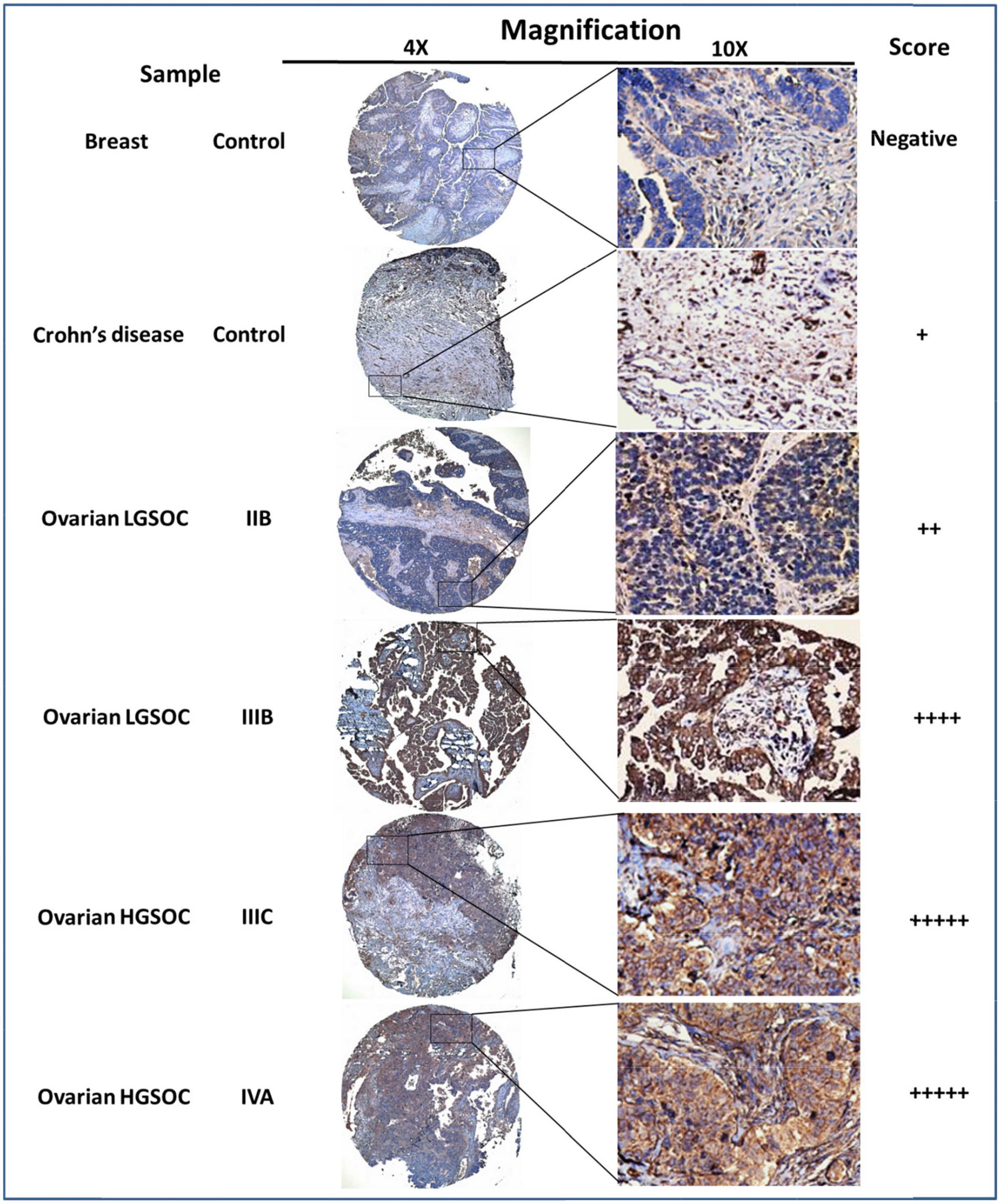

Supplementary Figure 2: ZIP4 was highly expressed in HGSOC tissues. Representative IHC images from different scores (Negative to ++++++) are shown.

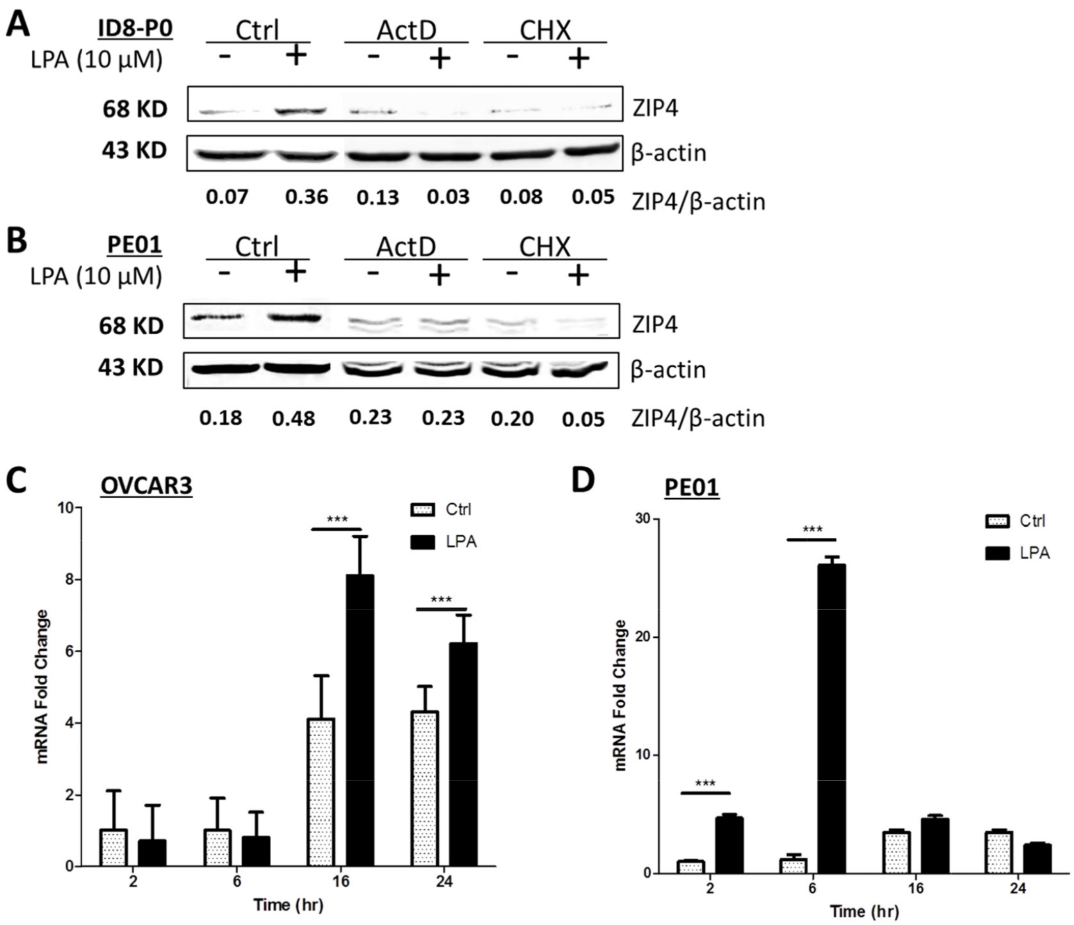

Supplementary Figure 3: LPA regulated ZIP4 expression at the transcriptional level. (A) LPA induced ZIP4 protein up-regulation expression (10 µM, 6 hr) in ID8-P0 cells was sensitive to both the transcriptional inhibitor ActD (1 µg/mL, 1 hr pre-treatment) and the translational inhibitor CHX (20 µg/mL, 1 hr pre-treatment). **(B)**. LPA-induced ZIP4 protein up-regulation by LPA (10 µM, 6 hr) analyzed by Western blot analyses. **(C-D)** ZIP4 mRNA levels were detected using Realtime-PCR in OVCAR3 and PE04 cells. Realtime RT-PCR conditions were described in Methods. $^{*}P < 0.05$; $^{**}P < 0.01$; and $^{***}P < 0.001$.

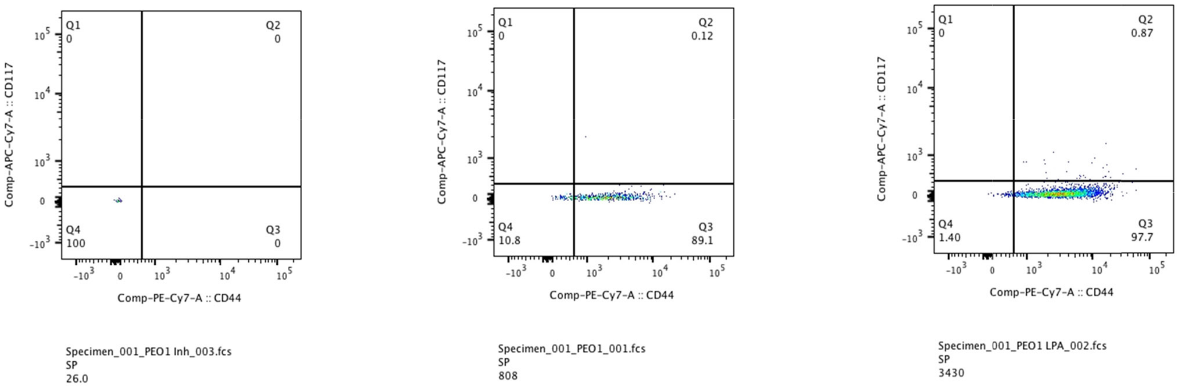

Supplementary Figure 4: LPA's effect on the CD44+CD117+ population. $CD44^{+}CD117^{+}$ cells were low (~ 0.12%), which was increased by LPA (10 µM, 6 hr treatment) to ~ 0.87% in SP population.
